# Supplementary figures and images for: Latent CMV infection of Lymphatic endothelial cells is sufficient to drive CD8 T cell memory inflation
Source: PLoS Pathog. 2023 Jan 23;19(1):e1010351. doi: 10.1371/journal.ppat.1010351 (PMC9894547; doi:10.1371/journal.ppat.1010351)

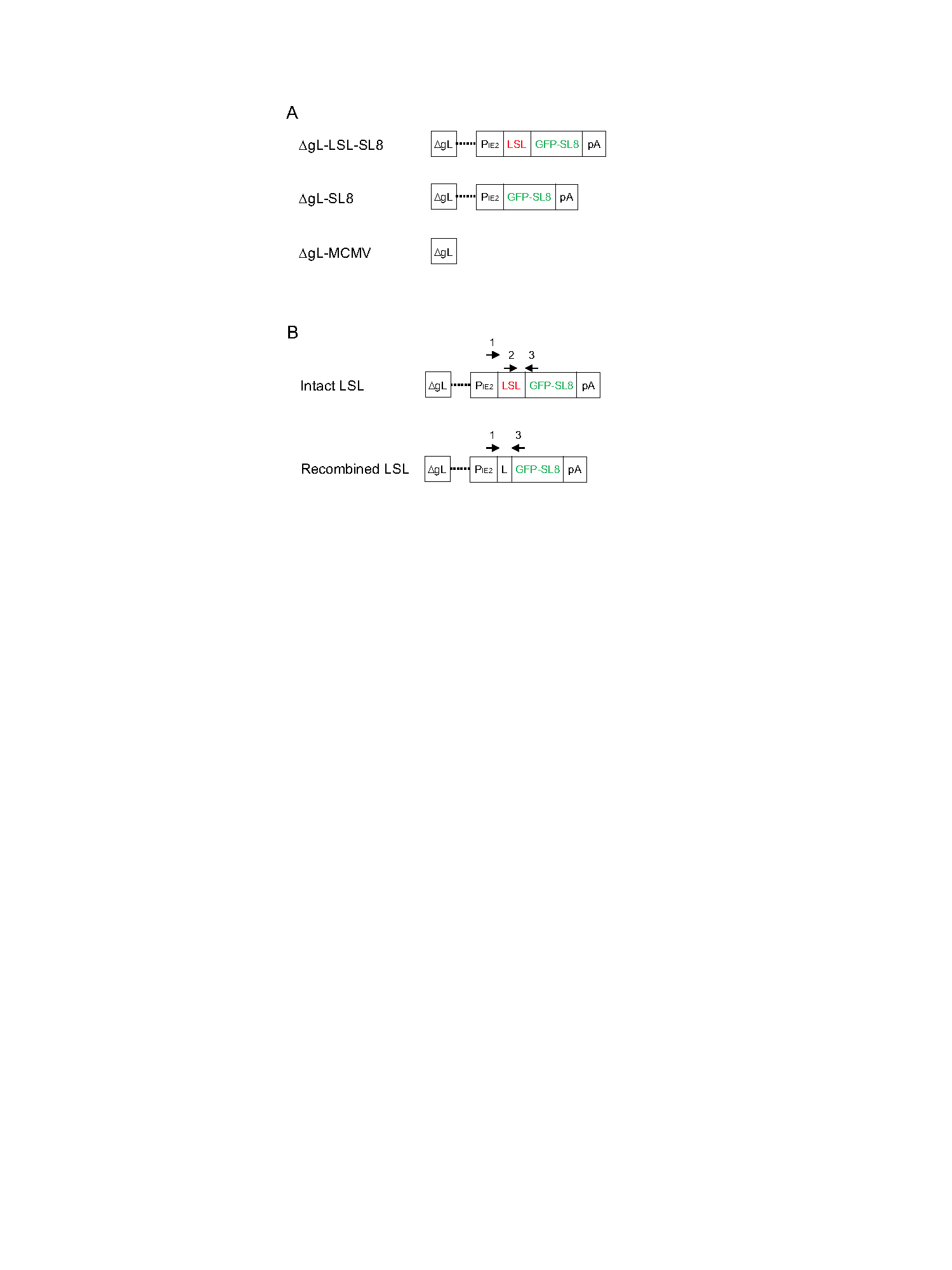

Supplement: S1 Fig — (A) Diagram of MCMV strains used in this study, all of which are grown on complementing gL-3T3 cells. ΔgL-LSL-SL8 MCMV has a lox-stop-lox (LSL) cassette which prevents constitutive expression of GFP with SL8 (SIINFEKL) linked to its C-terminus. GFP-SL8 is expressed after cre-dependent excision of the stop sequence. ΔgL-SL8 MCMV constitutively expresses GFP-SL8. ΔgL-MCMV lacks the gene encoding the essential glycoprotein gL. (B) Diagram of qPCR primer binding sites. When there is an intact LSL, primers 2 and 3 produce a ~100 bp amplicon. When there is a recombined LSL (i.e. a single loxP site), primers 1 and 3 produce a ~100 bp amplicon. (TIF) [file ppat.1010351.s001.tif]
